# Enhancing Prediction by Incorporating Entropy Loss in Volatility Forecasting

**DOI:** 10.3390/e27080806

**Published:** 2025-07-28

**Authors:** Renaldas Urniezius, Rytis Petrauskas, Vygandas Vaitkus, Javid Karimov, Kestutis Brazauskas, Jolanta Repsyte, Egle Kacerauskiene, Torsten Harms, Jovita Dargiene, Darius Ezerskis

**Affiliations:** 1 Department of Automation, Kaunas University of Technology, Studentu St. 48, 51367 Kaunas, Lithuania; 2 Faculty of Electrical and Electronics Engineering, Kaunas University of Technology, Studentu St. 48, 51367 Kaunas, Lithuania; 3 Fakultät Wirtschaft, Duale Hochschule Baden-Württemberg, Erzbergerstraße 121, 76133 Karlsruhe, Germany; 4 Higher Education Institution, Kauno Kolegija, Pramones pr. 20, 50468 Kaunas, Lithuania

**Keywords:** volatility forecasting, entropy loss function, realized volatility, HAR, HAR-RV, HARQ

## Abstract

In this paper, we propose examining Heterogeneous Autoregressive (HAR) models using five different estimation techniques and four different estimation horizons to decide which performs better in terms of forecasting accuracy. Several different estimators are used to determine the coefficients of three selected HAR-type models. Furthermore, model lags, calculated using 5 min intraday data from the Standard & Poor’s 500 (SPX) index and the Chicago Board Options Exchange Volatility (VIX) index as the sole exogenous variable, enrich the models. For comparison and evaluation of the experimental results, we use three metrics: Quasi-Likelihood (QLIKE), Mean Absolute Error (MAE), and Mean Squared Error (MSE). An empirical study reveals that the Entropy Loss Function consistently achieves the best QLIKE results in all the horizons, especially in the weekly horizon. On the other hand, the performance of the Robust Linear Model implies that it can provide an alternative to the Entropy Loss Function when considering the results of the MAE and MSE metrics. Moreover, research shows that adding more informative lags, such as Realized Quarticity for the Heterogeneous Autoregressive model yielding the Realized Quarticity (HARQ) model, and incorporating the VIX index further improve the general results of the models. The results of the proposed Entropy Loss Function and Robust Linear Model suggest that they successfully achieve significant forecasting accuracy for HAR models across multiple forecasting horizons.

## 1. Introduction

Volatility is the quality or state of being likely to change suddenly [1]. In finance, it is often referred to as the variance or standard deviation of an asset’s returns over a given period. As markets have become more complex and information-driven, accurate forecasting of volatility has become one of the main focuses of academics and practitioners [2]. Since the volatility depends on the market, macroeconomic conditions, financial leverage, and trading activity, it is often used to make financial decisions, such as asset allocation, market timing, derivative pricing, and risk evaluation over time [3]. As a result, researchers have developed models to forecast volatility and explore alternative ways to measure it, including range-based estimators that use high–low price ranges rather than returns [4].

In the second half of the 20th century, scholars began developing models that better captured the dynamic behavior of returns and volatility. Early theoretical works emphasized the importance of accurate volatility estimation, as seen in the Black–Scholes model [5], where volatility is critical in pricing derivative securities and managing financial risk. The introduction of Black–Scholes stimulated interest in alternative approaches to volatility modeling. A foundational breakthrough came with the development of Engle’s Autoregressive Conditional Heteroskedasticity (ARCH) model, which became the basis for many subsequent studies on volatility forecasting [6]. Further, Bollerslev introduced the Generalized Autoregressive Conditional Heteroskedasticity (GARCH) model, which extended the ARCH framework by incorporating a more flexible lag structure for past variances [7]. The later introduced Exponential Generalized Autoregressive Conditional Heteroskedasticity (EGARCH) approach [8] has a few advantages over GARCH. The first is that EGARCH measures the log returns, resulting in positive conditional variance. The second is that this model’s asymmetry allows for the capture of the leverage effect [9]. Glosten et al. introduced the Glosten–Jagannathan–Runkle Generalized Autoregressive Conditional Heteroskedasticity (GJR-GARHC) model, which improved the GARCH version by evaluating an additional variable, allowing for the capture of possible asymmetries [9,10]. Despite many claims that ARCH and stochastic models provide poor forecasting, the results of [11] contradict such a statement. The availability of intraday data has enabled the use of realized volatility to examine the stochastic properties of asset returns [12]. Building on stochastic volatility frameworks, research has revealed that volatility exhibits rough, fractal-like behavior rather than smooth diffusion. A later study, completed by Poon and Granger, concluded that GARCH models were dominating ARCH models in volatility forecasting [13]. Recently, Gatheral et al. proposed the Rough Volatility model, in which volatility followed a fractional Brownian motion with a Hurst exponent below 0.5, offering improved empirical fit and theoretical insights compared to classical stochastic volatility (SV) models [14]. The following study by Ulugbode and Shittu proposed a Transition EGARCH model that outperformed standard GARCH-type models in forecasting the conditional volatility of the Nigerian Stock Exchange. However, the authors also emphasized that more complex nonlinear models were not inferior to simpler alternatives and may provide greater flexibility in capturing market dynamics [15].

Over time, it became apparent that standard volatility models were insufficient to estimate daily volatility, especially over more extended periods, despite the available data. The increased quantities of available data allowed for a reduction in prediction horizons, shifting from quarterly or monthly to weekly or daily forecasts. Andersen et al. developed a method for modeling and forecasting the realized volatility and correlation. Researchers specified and estimated a long-memory Gaussian Value-at-Risk (VaR) model for daily realized volatilities. The proposed model successfully forecasted volatility and surpassed the GARCH model [16]. Later, Corsi proposed the Heterogeneous Autoregressive model of Realized Volatility (HAR-RV), which effectively captured long-memory properties in volatility. The model consistently outperformed short-memory alternatives across various forecasting horizons, including one day, one week, and two weeks [17]. Further, Andersen et al. proposed a new Heterogeneous Autoregressive model of Realized Variance with Continuous and Jump components (HAR-RV-CJ) forecasting model that supplemented the HAR-RV model, incorporating the discontinuous jumps to better capture the whole structure of asset return variability [18]. Afterward, research indicated that jump variation was important in predicting future volatility—negative jumps resulted in higher volatility and vice versa [19]. However, Prokopczuk et al. [20] found that explicit inclusion of jumps did not improve forecasting accuracy after comparing various HAR-RV model extensions over multiple forecasting periods. Despite the widespread application of GARCH-type models, recent studies suggest that realized and implied volatility measures contain valuable forward-looking information and can significantly enhance the forecasting accuracy of GARCH-based models [21]. Therefore, researchers have proposed simple yet effective forecasting models based on realized volatility (RV), which explicitly account for temporal variation in forecast errors. One such model is the HARQ model, an extension of the HAR framework that incorporates error-based adjustments to enhance forecast accuracy [22]. To better account for regime-switching behavior and nonlinear dynamics in electricity markets, recent extensions of the HAR-RV model, such as the Logistic Smooth Transition HAR (LST-HAR) framework, have been proposed, showing improved forecasting accuracy over traditional linear HAR specifications [23]. To address cross-asset volatility dynamics, Cubadda and Hecq [24] proposed a Vector HAR (V-HAR) index model, which captured standard volatility components across multiple assets and improved forecast performance in multivariate settings. Later, Clements and Preve suggested an approach to enhance volatility forecasting by supplementing HAR models with Ridge Regression (RR) and simple Weighted Least Squares (WLS) techniques [25]. Furthermore, Li et al. extended the HAR model by incorporating over 200 cross-market predictors into a shrinkage framework using Least Absolute Shrinkage and Selection Operator (LASSO) and Elastic Net techniques. Investigation results showed that integrating global realized volatility components, jump measures, and uncertainty indices significantly improved forecasting accuracy [26]. Recently, Michael et al. augmented the HAR model with a range of volatility estimators by applying dimensionality reduction techniques to implied volatility surfaces and calibrating stochastic volatility model parameters, including Heston and Bates, to extract implied volatility estimators. This analysis indicated that these augmentations significantly enhanced daily realized volatility forecasting and could effectively improve VIX prediction [27].

The need for improved volatility forecasting and the development of computing capabilities led to the investigation and development of advanced methods, such as machine learning (ML) and Artificial Neural Networks (ANNs). Therefore, Luong and Dokuchaev introduced the Random Forest (RF) algorithm for forecasting realized volatility, suggesting that using purified implied volatility as an input and applying the RF technique significantly enhanced the predictive performance of the traditional HAR model [28]. Later on, Bucci demonstrated that feedforward neural networks could outperform traditional volatility forecasting models by effectively capturing complex nonlinear dynamics and structural breaks in financial time series. Among the models evaluated, Long Short-Term Memory (LSTM) and Nonlinear Autoregressive with Exogenous Input (NARX) delivered the most accurate realized volatility forecasts [29]. Zhang et al. proposed the use of Temporal Convolutional Networks (TCNs) for forecasting stock volatility and Value-at-Risk (VaR), with the empirical results indicating superiority of this model over the traditional GARCH-type models in forecasting both volatility and downside risk [30]. However, ML approaches often encounter challenges, as expressed by Ge et al., who systematically reviewed studies published since 2015 on neural network-based approaches for financial volatility forecasting, describing the difficulty in directly comparing model performance across studies and the gap between modern and standard ML techniques [31]. Further, Zahid and Saleem investigated the performance of Support Vector Machine (SVM) models under high-volatility conditions during the COVID-19 pandemic, revealing that the Radial Basis Function (RBF) kernel outperformed both linear and polynomial kernels [32]. As discussed by Christensen et al., neural networks are superior to detecting nonlinearity in market dynamics and extracting valuable information related to implied volatility, especially over longer forecasting horizons [33]. Therefore, Souto and Moradi introduced the Neural Basis Expansion Analysis for Time Series with Exogenous Variables (NBEATSx) model for realized volatility forecasting, demonstrating its superior predictive accuracy across multiple stock indices when compared to both traditional econometric models, such as HAR and GARCH, and advanced deep learning architectures, including LSTM and Temporal Convolutional Networks (TCNs) [34]. Additionally, Zhang et al. proposed a machine learning approach that leveraged intraday volatility commonality across multiple assets to enhance forecasting accuracy, and another approach to forecast the coming-day RV by using past intraday RVs as a predictor. The latter proposed approach yielded a superior out-of-sample forecast compared with traditional models [35]. Recently, multiple researchers have noticed that the application of machine learning (ML) methods to capture nonlinear dynamics and complex structures often delivers far better volatility forecasting results than traditional econometric models [36]. The study by Lolic indicated that Random Forest and Gradient Boosting models consistently outperformed traditional econometric models [37]. Beg analyzed machine learning models for volatility forecasting and revealed that Random Forest (RF) consistently delivered the most accurate predictions and high correlation with the observed data. On the other hand, neural network or Gradient Boosting models performed moderately, requiring extensive tuning for optimal results. Linear regression and Support Vector Regression (SVR) yielded the weakest forecasts due to their limited ability to capture nonlinear dependencies in financial data [38]. The study by Mansilla-Lopez et al. concluded that machine learning models consistently outperformed traditional econometric approaches in forecasting financial market volatility. The authors proposed a unifying definition of volatility by comparing various statements in different articles: “volatility is an indicator of market risk, which measures the variation in the returns of a financial asset over a period of time” [39].

Recent research has introduced hybrid models that integrate GARCH-family structures with neural networks, leveraging the strengths of both econometric and machine learning approaches. Following the desire to develop hybrid models, Monfared and Enke applied different ML methods to volatility forecasting. The results showed improvement for extreme event forecasting, but the authors did not suggest using a hybrid approach for low-volatility periods due to the complexity of the model [40]. Implementing Artificial Neural Networks (ANNs) alongside GARCH models improved forecast accuracy by over 10% when applied to Latin American financial markets, demonstrating the effectiveness of hybrid methodologies in emerging market contexts [41]. Yang et al. introduced another approach, employing hybrid modeling by integrating the Support Vector Machine (SVM) algorithm within a big data framework. Their results demonstrated that effective feature selection and dimensionality reduction significantly enhanced prediction accuracy and computational efficiency in large-scale volatility forecasting tasks [42]. Later, Trierweiler Ribeiro et al. integrated the Heterogeneous Autoregressive (HAR) framework with an Echo State Neural Network (ESN) and Particle Swarm Optimization (PSO), leveraging different strengths from each method. The HAR-ESN-PSO model demonstrated an increased predictive accuracy across multiple forecasting horizons [43]. Liu et al. combined Bidirectional Recurrent Neural Networks (Bi-RNNs), Gated Recurrent Units (GRUs), and Particle Swarm Optimization (PSO). The proposed GBP (GRU–BiRNN–PSO) model showed increased forecasting performance across several datasets, thus demonstrating improved learning capacity and generalization ability compared to traditional methods [44]. The same year, Mishra et al. introduced a more accurate hybrid model integrating GARCH and LSTM components within a bagged attention mechanism. The Multi-Task Generalized Autoregressive Conditional Heteroskedasticity (MT-GARCH) and Multi-Task Learning Generalized (MTL-GARCH) models demonstrated superior predictive accuracy and robust risk estimates across diverse markets during elevated volatility windows, such as the COVID-19 pandemic [45]. Additionally, Brini and Toscano introduced SpotV2Net, an NN with a Graph Attention Network (GAT) architecture for multivariate intraday spot volatility forecasting. The model captured dynamic cross-asset dependencies and spillover effects, delivering significant gains in prediction accuracy compared to traditional econometric and alternative machine learning models [46]. Most recently, Hu et al. combined Convolutional Neural Networks (CNNs) with the Heterogeneous Autoregressive–Kitchen Sink (HAR-KS) to forecast the direction of stock market volatility. Empirical results from the Chinese stock market showed that the CNN-HAR-KS model outperformed traditional econometric and standalone deep learning models in forecasting performance [47]. Furthermore, Li et al. augmented the classical HAR-RV and LSTM models with additional influencing factors. The results showed that while factor integration improved the performance of both models, the LSTM consistently outperformed HAR-RV. Furthermore, incorporating Principal Component Analysis (PCA) into the LSTM architecture yielded the highest forecasting accuracy across all experimental configurations [48]. Finally, Kumar et al. combined Variational Mode Decomposition (VMD) with deep learning models, including ANN, LSTM, and GRU. The proposed Q-VMD-ANN-LSTM-GRU approach achieved satisfactory forecasting results across multiple stock indices, demonstrating strong potential for improving financial risk management, stress testing, and investment strategy formulation [49].

Researchers have increasingly recognized that effective volatility forecasting requires more than just historical price data or directional predictions; it also demands attention to the magnitude of price movements and the integration of public sentiment with macroeconomic indicators. To address this need, Wang et al. proposed the Economics (ECON) framework, which combined filtered tweet sentiments, government-derived macroeconomic statistics, and historical price data to forecast stock movement and volatility together. The ECON model significantly improved predictive accuracy by modeling various relationships between individual stocks, industry sectors, and macroeconomic conditions [50]. Consequentially, Shi et al. introduced a novel framework for volatility forecasting by developing NumBERT, a pre-trained language model specifically designed to enhance the interpretation of numerical information in earnings call transcripts. Their approach improves contextual understanding of financial text and significantly increases the accuracy of 3-day volatility predictions [51]. The following study by Li et al. developed a Hierarchical Transformer-based model to forecast asset volatility by extracting risk information from annual reports. The authors constructed investment portfolios using Natural Language Processing (NLP), which successfully predicted beta values that outperformed the SPX by an average of 21 %. The results of these studies demonstrated the potential of deep language models in volatility-aware asset management strategies [52].

**Figure 1:**
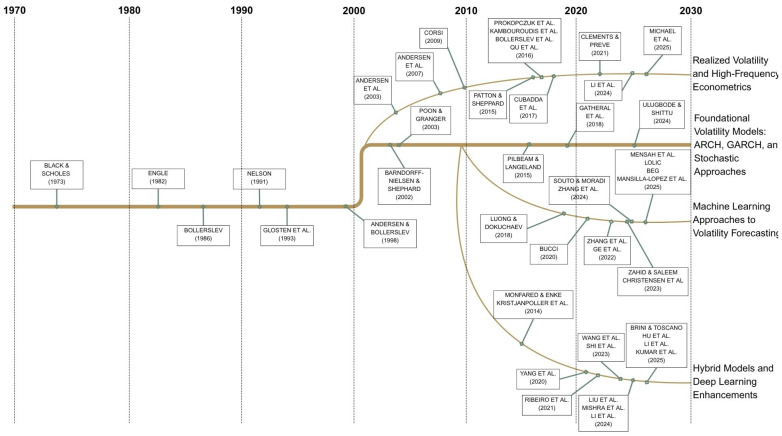
Development of volatility forecasting models [5,6,7,8,9,10,11,12,13,14,15,16,17,18,19,20,21,22,23,24,25,26,27,28,29,30,31,32,33,34,35,36,37,38,39,40,41,42,43,44,45,46,47,48,49,50,51,52].

This analysis of the literature shows the progression and shift from traditional econometrics to advanced forecasting methods, such as employing machine learning or using hybrid approaches (see Figure 1). This research paper proposes to take an additional step and presents a novel methodology for enhancing volatility forecasting by implementing entropy loss. The proposed method is based on the HAR-RV model, which is widely used in the field of volatility forecasting. The HAR-RV model captures the long-memory properties of volatility and has been shown to outperform other models in various studies. By incorporating entropy loss into the HAR-RV framework, we aim to improve the accuracy of volatility predictions. To verify the proposed method, a large dataset of high-frequency SPX data covering the period from 2 January 2008 to 15 April 2025 is used. The results demonstrate that the HAR-RV model with entropy loss significantly outperforms traditional HAR-RV models regarding forecasting accuracy. This research contributes to the existing literature by providing a novel approach to volatility forecasting that combines the strengths of HAR-RV models with the benefits of entropy loss.

This paper is organized as follows: Section 2 introduces the methodology and development of the proposed method. Section 3 presents the investigation’s results and discussions. Finally, Section 4 concludes the whole article.

## 2. Materials and Methods

### 2.1. Data Description

Table 1 provides a sample of the high-frequency dataset used in this study. The data, obtained under a licensed agreement from “First-rate Data”, consisted of five variables: Datetime, Open, High, Low, and Close prices. Each observation corresponded to a distinct 5 min interval within standard U.S. stock market trading hours. For every time interval, the dataset recorded the opening price, the highest and lowest prices observed during the interval, and the closing price. The following list provides definitions for each variable.

- Datetime: indicating the timestamp of the 5 min subinterval of the values (e.g., “2008-01-02 09:35:00”);
- Open: the opening price at the beginning of a 5 min subinterval (e.g., “1470.17”);
- High: the highest price reached within a 5 min subinterval (e.g., “1470.17”);
- Low: the lowest price reached within a 5 min subinterval (e.g., “1467.88”);
- Close: the closing price at the end of the 5 min subinterval (e.g., “1469.49”).

The intraday (5 min interval) SPX dataset used in this study covered the period from 2 January 2008 to 15 April 2025 and contained a total of 3,467,665 5 min intervals, which was equal to approximately 4350 trading days.

**Figure 2:**
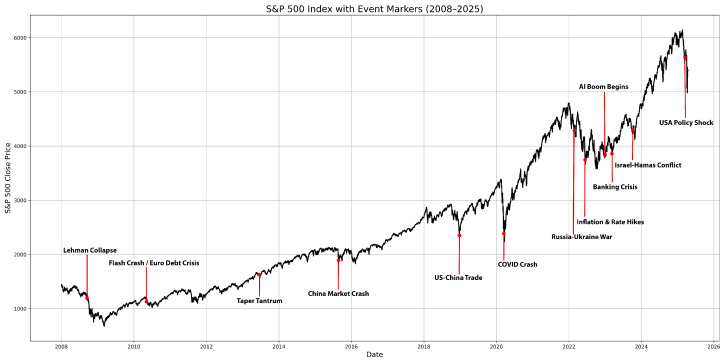
The evolution of the SPX index based on 5 min closing prices (P) since January 2008.

Figure 2 illustrates the evolution of the SPX 5 min closing price from 2008 to 2025, highlighting significant financial crisis periods and prolonged bull markets, as observed through high-frequency market data.

Several factors contributed to these changes, including supply and demand differences in the market. Overall, the primary factors were as follows:

- Last-minute updates or new information, such as macroeconomic announcements and earnings reports;
- Herding behavior or panic selling, plausibly caused by changes in investor sentiment [53];
- Changes in market liquidity and trading intensity reinforce price volatility, particularly during periods of stress [54].

Such factors make the modeling of time-dependent variance (volatility) a critical component in financial econometrics.

One of the primary reasons for using such extensive data is that to perform a detailed analysis, model evaluation should extend over longer periods that include periods of high and low volatility. The data used in this study contained the following major events that had a significant impact on the stock market and the SPX index between 2008 and 2025:

- The great financial crisis in 2008–2009.
- The European sovereign debt crisis in 2010–2012.
- The “Taper Tantrum” in 2013.
- The Chinese stock market turmoil and oil price crash in 2015–2016.
- The U.S.–China trade war in 2018.
- The COVID-19 pandemic in 2020.
- Inflation, aggressive rate hikes, and the Russia–Ukraine war in 2022. A combination of factors created a bear market for most of 2022:
  - High inflation;
  - Monetary tightening;
  - Russia–Ukraine conflict.
- The U.S. regional banking crisis and Israel–Hamas conflict in 2023.
- The Artificial Intelligence (AI) boom in 2023–2025.
- Weakening corporate outlook, political uncertainty, and Israel–Iran 12-day war in 2025.

Using intraday frequency data provided temporal granularity, a key component for constructing realized variance. Additionally, it provided detailed observations on long-term price evolution. As HAR-type models capture nonlinear, clustered, and shock-sensitive features of financial markets, the data frame was well suited for examining realized variance through a multi-horizon lag structure.

### 2.2. Return and Realized Measure Construction

Theoretically, volatility is an instantaneous variable. Hence, it is possible to study it in a continuous-time diffusive environment. Therefore, the focus was on a single financial asset and the process modeling of its logarithmic price (log $P_{t}$), assuming it continuously evolved in the market during trading hours. Furthermore, in this article, the closing price of a single asset at time *t* was denoted as *P*.

If $P_{t-1+i\Delta }$ is the closing price at the end of the *i*th subinterval, $P_{t-1+(i-1)\Delta }$ the price at the beginning of the *i*th subinterval, t−1 the start of the current day, *t* the end of the current day, Δ the time interval in fractions of a day, and *M* the total number of subintervals [25], then $r_{t,i}$, the logarithmic return for the *i*th subinterval during trading day *t*, can be expressed as Equation (1):

(1) $$\begin{array}{ccc} r_{t,i} & =\log P_{t-1+i\Delta }-\log P_{t-1+(i-1)\Delta }, & \;i=1,2,\ldots ,M\;, \end{array}$$

where the sampling frequency, M=1/Δ, is the number of subintervals in a day. For instance, with 5 min of data in a 6.5 h (390 min) trading day, this equals M=390/5=78. The choice of interval length affects the bias–variance trade-off in estimating the realized variance, as presented in Table 2. Empirical studies show that 5 min intervals generally minimize the Mean Squared Error when estimating daily variance using intraday returns [55].

Classical models such as Ordinary Least Squares (OLS) and many robust regressions often rely on assumptions of normally distributed errors. However, in high-frequency financial data, returns typically exhibit fat tails and volatility clustering, violating these assumptions and motivating the use of alternative or robust modeling techniques. While the classical HAR model estimated via OLS relies on assumptions that are violated in high-frequency financial data (e.g., normality, homoskedasticity), it remains widely used due to its relative success in forecasting realized variance. Nevertheless, robust and distribution-flexible extensions of HAR are adopted to address fat-tailed and heteroskedastic error structures in practice.

Researchers have discovered that logarithmic returns exhibit approximately Gaussian properties at intraday levels, making them preferable for volatility modeling [11,56]. One of the advantages of taking the logarithm of price differences is that it reduces skewness in the return distribution and helps to stabilize variance, especially in volatile market conditions. Logarithmic return transformation enhances the statistical properties of the data, making it a suitable input feature for regression-based models, such as OLS, WLS, and robust HAR variants. Thus, these estimation techniques become more reliable and less sensitive to outliers and sudden price jumps. Considering this, Equation (2) describes the daily logarithmic return for the active part of trading day *t*.

(2) $$r_{t}=\sum_{i=1}^{M}r_{t,i},$$

where $r_{t}$ is the total return for the day *t*, and *M* is the number of subintervals in a day.

**Figure 3:**
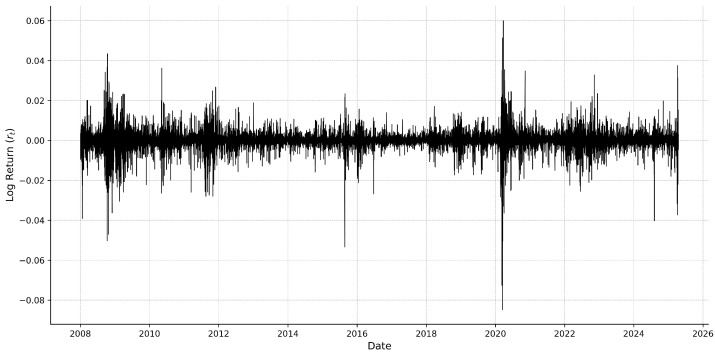
The 5 min log returns $\left(r_{t}\right)$ of the SPX index since January 2008.

Figure 3 illustrates how the SPX 5 min log returns change over the sample period. The series captures intraday return fluctuations over approximately 3.4 million intervals. The plot highlights several important empirical features that are commonly observed, such as volatility clustering in high-frequency financial data. The return values typically change around zero. The visible spikes reflect the crisis periods, such as the 2008 global financial crisis and the 2020 pandemic, among others. These spikes highlight the heavy-tailed and heteroskedastic character of return distributions by reflecting times of high uncertainty and abrupt price changes.

### 2.3. Stochastic Modeling of Log Price Dynamics

Before analyzing the dynamics of the logarithmic price process, it is essential to understand the underlying stochastic process that explains its evolution, namely, Brownian motion. Brownian motion, also known as the Wiener process, forms the foundation of continuous-time modeling in financial economics. In stochastic calculus, it is essential to the formulation of asset price dynamics. A standard Brownian motion is a continuous-time stochastic process $W_{t}$ with the following properties [57]:

- $W_{0}$ = 0—the starting point is always equal to zero.
- With probability 1, the function $t\to W_{t}$ is continuous over the period *t*.
- The process ${\left\{W_{t}\right\}}_{t\geq 0}$ has stationary, independent increments.
- The increment $W_{t+s}\to W_{s}$ follows a NORMAL (0 t) distribution.

A standard *d*-dimensional Wiener process is a vector-valued stochastic process that can be defined as Equation (3):

(3) $$W_{t}=\left(W_{t}^{\left(1\right)},W_{t}^{\left(2\right)},W_{t}^{\left(3\right)},\ldots ,W_{t}^{\left(d\right)}\right),$$

where components $W_{t}^{\left(i\right)}$ are independent, standard one-dimensional Wiener processes.

Determining how the logarithm of the asset price changes over time is one of the fundamental problems in financial asset price modeling. The majority of forecasting and volatility estimation techniques, including the HAR family of models, make forecasts based on hypotheses of a continuous-time stochastic process that underlies returns. The conventional constant-volatility model and the more realistic stochastic volatility (SV) framework are the two main modeling frameworks. These models differ in their handling of return variance, which has important ramifications for both the empirical validity of financial models and the precision of volatility predictions.

#### 2.3.1. Classical Approach: Geometric Brownian Motion (GBM)

**Figure 4:**
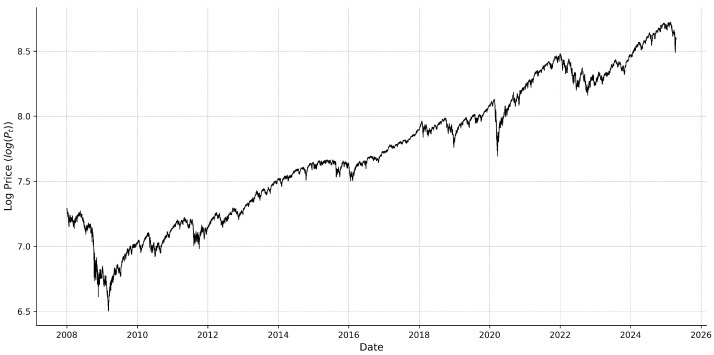
SPX intraday 5 min $\left(\log P_{t}\right)$ over time.

Equation (4) describes how the log of the price (*P*) of a single asset changes continuously over time during the trading day:

(4) $$d\log\left(P_{t}\right)=\mu_{t}dt+\sigma_{t}dW_{t},$$

where μdt is the instantaneous drift term, which represents the expected direction of the logarithmic price. $\sigma_{t}=\sqrt{\sum r_{t}^{2}}$ is the volatility process that inflates the price change concerning $W_{t}$, and *W* is a standard Brownian motion. $\sigma_{t}\;$d$W_{t}$ is the random movement of prices where volatility comes from. We can see the change in the log price $\left(\log\left(P_{t}\right)\right)$ in Figure 4.

Due to its closed form and ease of analysis, this formulation has long been a cornerstone of financial theory and frequently linked to the Black–Scholes option pricing model. Nevertheless, it assumes that volatility is always constant, with normally distributed returns, which do not accurately reflect practical scenarios [58]. Thus, in empirical finance, these assumptions are considered oversimplified. The classic GBM framework poorly captures such behaviors in actual markets that exhibit volatility clustering, leptokurtosis (fat tails), and time-varying risk [59]. Following this, Equation (5) defines $W_{t}$ for the current application:

(5) $$dW_{t}=\frac{d\log\left(P_{t}\right)-\mu_{t}dt}{\sigma_{t}},$$

where $dW_{t}$ is the increment in the Brownian motion process, $d\log(P_{t})$ is the change in the logarithm of the asset return price, $\mu_{t}$ is the instantaneous drift term, and $\sigma_{t}$ is the instantaneous volatility. Given the use of high-frequency intraday data, the instantaneous drift component $\mu_{t}dt$ in Equation (4) becomes negligible in comparison to the variance-driven component of price evolution. Both the theoretical and empirical literature support this assumption [11], resulting in the reduction of Equation (5) to Equation (6), which emphasizes that the underlying Brownian motion $W_{t}$, scaled by instantaneous volatility $\sigma_{t}$, can be represented by normalized log price increments:

(6) $$dW_{t}=\frac{d\log(P_{t})}{\sigma_{t}}$$

#### 2.3.2. Generalized Approach: Stochastic Volatility Models

To overcome the limitations of the classic GBM approach, researchers have developed stochastic volatility models that simulate variance as a stochastic process rather than a fixed constant. Barndorff-Nielsen and Shephard notably proposed Equation (7) in [12]:

(7) $$dy^{*}\left(t\right)=\left\{\mu +\beta \sigma^{2}\left(t\right)\right\}dt+\sigma \left(t\right)\;dw\left(t\right),$$

where $y^{*}\left(t\right)=\log\left(P_{t}\right)$ is the logarithmic price; μ is the drift term, representing the expected rate of change in the logarithmic price; $\sigma^{2}\left(t\right)$ is the instantaneous variance that evolves stochastically over time; β reflects a risk premium component that increases the drift in proportion to the current level of variance; and dw(t) is a standard Brownian motion. σ(t) is the instantaneous (spot) volatility, modeled as a latent stochastic process that evolves continuously over time. Unlike constant-volatility models, the SV framework allows $\sigma^{2}\left(t\right)$ to change in response to shocks, mean-revert, or persistent behavior, which reflects actual market dynamics even more accurately. The stochastic volatility approach addresses some key empirical features that the classic GBM approach is unable to capture, such as volatility clustering. Additionally, the inclusion of $\beta \sigma^{2}\left(t\right)$ in the drift term introduces a risk–return trade-off, which allows expected returns to change proportionally to volatility. By allowing stochastically variant changes, the model is now capable of replicating heavy tails. Barndorff-Nielsen and Shephard defined the SV model over small intervals as Equation (8) [12]:

(8) $$y_{i}=y^{*}\left(i\Delta \right)-y^{*}\left((i-1)\Delta \right),$$

where $y_{i}$ is the change in the logarithmic price over the *i*th subinterval of length Δ during the trading day. This approach is equivalent to Equation (9), assuming that each return is conditionally normally distributed, as per the SV model:

(9) $$r_{t,i}|\sigma_{t,i}^{2}∼N\left(\mu_{i},\sigma_{t,i}^{2}\right),$$

where $r_{t,i}$ is the return for the *i*th subinterval of day *t*, $\sigma_{t,i}^{2}$ is the instantaneous variance at that subinterval, and $\mu_{i}$ is the drift term for that subinterval. The actual latent volatility of the asset at time *t* is not directly observable. Instead, the objective is to estimate the total variation in volatility accumulated over a day, known as the integrated variance (IV) (see Equation (10)).

(10) $$IV_{t}=\int_{t-1}^{1}\sigma^{2}\left(t_{s}\right)dt_{s},$$

where $IV_{t}$ is the integrated variance for trading day *t*, and $\sigma^{2}\left(t_{s}\right)$ is the instantaneous variance at time $t_{s}$ within that day. This quantity captures the total uncertainty or risk in asset returns for day *t*. It is the theoretical limit of the sum of intraday return variances as the sampling frequency increases. In volatility models such as HAR or HARQ, the instantaneous drift $\left(\mu_{t}\right)$ is not the focus—the randomness or volatility part is, because the drift is slight compared to volatility at high frequencies. A key insight from stochastic volatility (SV) theory is that the integrated variance over a given time interval can be recovered directly from the sample path of the log price process.

In reality, the instantaneous variance is unobservable due to the discrete and noisy nature of market data, analogous to measuring continuous stochastic fields with limited-resolution instruments in statistical physics. This idea parallels projection methods in nonequilibrium systems and has been formalized in financial econometrics by [12,60], echoing techniques developed in statistical physics by [61]. Moreover, observed high-frequency returns nonparametrically estimate the integrated variance without requiring knowledge of the drift (μ) or the precise form of the stochastic process governing σ(t). It is model-free regarding the volatility dynamics and is consistent as long as the sampling frequency is sufficiently high. One way to estimate it is by using realized variance (RV), as shown in Equation (11):

(11) $$\begin{array}{ccc} RV_{t} & =\sum_{i=1}^{M}r_{t,i}^{2}\approx IV_{t}=\int_{t-\Delta t}^{t}\sigma^{2}\left(t_{s}\right)dt_{s},\; & \mathrm{as}\;M\to \infty \;\mathrm{or}\;\Delta t\to 0, \end{array}$$

where $RV_{t}$ is the realized variance for trading day *t*, $r_{t,i}$ is the return for the *i*th subinterval of day *t*, and Δt is the length of the subinterval in fractions of a day. Realized variance in the trading day *t* can be defined as the sum of the squared returns. The realized variance (RV) is a reasonable estimate of the actual volatility over a day using high-frequency data. This connection is foundational to the HAR-RV framework and the use of high-frequency data in volatility forecasting. Nevertheless, even then, there is some error in that estimate (see Equation (12)). Under certain conditions on *M*, the sampling frequency and estimation error $\eta_{t}$ defined in [12] can be determined using Equation (13):

(12) $$RV_{t}=IV_{t}+\eta ,$$

(13) $$\frac{\eta_{t}}{\sqrt{(}2\Delta IQ_{t})}=\frac{RV_{t}-IV_{t}}{\sqrt{(}2\Delta IQ_{t})};$$

where $\eta_{t}$ is the estimation error, $RV_{t}$ is the realized variance for trading day *t*, $IV_{t}$ is the integrated variance for trading day *t*, and $IQ_{t}$ is the integrated quarticity for trading day *t*.

Assuming the error term is scaled by the integrated quarticity, its form follows a standard normal distribution, N(0,1). This normalization implies that realized quarticity (RQ) can serve as an estimator of integrated quarticity (IQ). In this context, Equations (14) and (15) formalize the definitions of RQ and IQ, respectively.

(14) $$RQ_{t}=\frac{M}{3}\sum_{i=1}^{M}r_{t,i}^{4},$$

where $RQ_{t}$ is the realized quarticity for trading day *t*, and $r_{t,i}$ is the return for the *i*th subinterval of day *t*. The factor $\frac{M}{3}$ is a scaling factor.

(15) $$IQ_{t}=\int_{t-1}^{t}\sigma_{s}^{4}ds,$$

where $IQ_{t}$ is the integrated quarticity for trading day *t*, and $\sigma_{s}^{4}$ is the fourth power of the instantaneous volatility at time *s*. As the sampling frequency increases, RV becomes closer to IV, but the estimation error ($\eta_{t}$) remains. Equation (12) scales the estimator error because it is directly uninterpretable. To statistically interpret this error meaningfully, it should be differently scaled, which leads to results with a known distribution and allows for confidence intervals.

The incorporation of higher-order moments, such as integrated quarticity, has been used for refining the statistical properties of realized measures and addressing the scaling of estimation errors. In light of this, attention is next directed toward the Heterogeneous Autoregressive (HAR) class of models, wherein realized volatility components across multiple horizons are utilized for modeling and forecasting return variance.

### 2.4. Heterogeneous Autoregressive-Type Model Specifications

This section presents the Heterogeneous Autoregressive (HAR) model, adopted in this study as the baseline model and originally proposed by [17]. Being a pioneering study, Corsi’s work demonstrated that if realized variance was modeled on the basis of multiple heterogeneous time horizons, it would be possible to effectively account for long-memory and volatility persistence in financial markets. According to this setup, this research adds to the original HAR model specifications by including realized quarticity and the VIX index as an external variable that is forward-looking in terms of market expectations. These modifications are intended to improve the model’s predictive power, particularly when uncertainty is heightened.

#### 2.4.1. Heterogeneous Autoregressive Model of Realized Volatility

Since high-frequency intraday data are widely available, current researchers have considered using realized volatility to create forecasting models for the time-varying return volatility. Because of its ease of use and reliable prediction results, the HAR model has become one of the most popular forecasting models in practical applications. Corsi first introduced the HAR model [17], derived from a simple expansion of heterogeneous models such as ARCH or HARCH examined by Müller et al. [62]. To parameterize the conditional variance of the discretely sampled returns, this method uses the squared returns across longer and/or shorter forecasting horizons, along with the delayed squared returns over the same horizon. Assuming a typical trade week and month are 5 and 22 days, respectively, as a linear function of the daily, weekly, and monthly realized variance components, RV is specified in the original HAR model and defined as Equation (16):

(16) $$\begin{array}{ccc} RV_{t} & =\beta_{0}+\beta_{1}RV_{t-1}^{\left(d\right)}+\beta_{2}RV_{t-1}^{\left(w\right)}+\beta_{3}RV_{t-1}^{\left(m\right)}+e_{t}\; & \;\left\{\begin{array}{c} RV_{t-1}^{\left(d\right)}\equiv RV_{t-1},\\ RV_{t-1}^{\left(w\right)}\equiv \frac{1}{5}\sum_{i=1}^{5}RV_{t-i},\\ RV_{t-1}^{\left(m\right)}\equiv \frac{1}{22}\sum_{i=1}^{22}RV_{t-i}, \end{array}\right. \end{array}$$

where $RV_{t}$ is the realized variance of a trade day *t*, and $RV_{t-1}^{\left(d\right)}$, $RV_{t-1}^{\left(w\right)}$, and $RV_{t-1}^{\left(m\right)}$ are the daily, weekly, and monthly realized variances at time t−1, respectively. The β coefficients are the parameters to be estimated, and $e_{t}$ is the zero mean error term. This RV definition parsimoniously captures the strong persistence seen in most realized variance series.

#### 2.4.2. Heterogeneous Autoregressive Model of Realized Quarticity

Realized Quarticity (RQ) provides a deeper insight into the dynamics of the volatility process. While Realized Variance (RV) measures the magnitude of price movements, RQ quantifies the “volatility of volatility.” It shows how stable the overall market risk is. A high RQ suggests that the level of risk is not only elevated but also highly unstable and prone to sudden jumps. By incorporating this measure, the HARQ model is expected to gain forecasting advantages. The primary benefit is the model’s enhanced ability to adapt to changing market structures, allowing it to better distinguish between periods of high but persistent volatility and periods where the variance process itself is erratic. This adaptiveness is anticipated to yield more accurate and reliable forecasts, particularly during episodes of market stress or significant structural shifts. Bollerslev et al. proposed the extended HAR by (typically) estimating it with OLS and considering the error that occurs from RV estimation by employing Realized Quarticity, which gave the model its name, HARQ [22]. Equation (17) describes the extended HAR:

(17) $$\begin{array}{cc} RV_{t} & =\beta_{0}+\left(\beta_{1}+\beta_{1Q}\sqrt{RQ_{t-1}^{\left(d\right)}}\right)RV_{t-1}^{\left(d\right)}+\left(\beta_{2}+\beta_{2Q}\sqrt{RQ_{t-1}^{\left(w\right)}}\right)RV_{t-1}^{\left(w\right)}\\ \; & +\left(\beta_{3}+\beta_{3Q}\sqrt{RQ_{t-1}^{\left(m\right)}}\right)RV_{t-1}^{\left(m\right)}+e_{t}, \end{array}$$

where $RQ_{t-1}^{\left(d\right)}$, $RQ_{t-1}^{\left(w\right)}$, and $RQ_{t-1}^{\left(m\right)}$ are the daily, weekly, and monthly realized quadratic lags at time t−1, respectively. The β coefficients are the parameters to be estimated, and $e_{t}$ is the zero mean error term. For short-term forecasting, Bollerslev proposed using the daily lag, as described by Equation (18). Such a change in the equation is helpful since the estimation error is primarily responsible for the attenuation bias in the predictions (RV is less persistent than IV). When the measurement uncertainty recorded by RQ is larger, this paradigm reduces the weight of the past RV observations:

(18) $$\begin{array}{cc} RV_{t} & =\beta_{0}+\left(\beta_{1}+\beta_{1Q}\sqrt{RQ_{t-1}^{\left(d\right)}}\right)RV_{t-1}^{\left(d\right)}+\beta_{2}RV_{t-1}^{\left(w\right)}+\beta_{3}RV_{t-1}^{\left(m\right)}+e_{t}, \end{array}$$

where $RQ_{t-1}^{\left(d\right)}$ is the daily realized quadratic lag at time t−1. The β coefficients are the parameters to be estimated, and $e_{t}$ is the zero mean error term.

Exogenous variables can further extend the HARQ model. One such variable is VIX, which stands for the Volatility Index and gauges the market’s expectation of future volatility over the coming 30 days. Sometimes, others refer to this index as the “fear index” since it rises as investors anticipate market uncertainty or stability. The Chicago Board Options Exchange (CBOE) determines this index from the S&P 500 Index option prices. It reflects the implied volatility of S&P 500 index options and does not consider the past volatility as RV. The introduction of VIX to the HARQ model results in the Heterogeneous Autoregressive Model with Realized Quarticity and Exogenous Variables (HARQ-X) model expressed in Equation (19):

(19) $$\begin{array}{cc} RV_{t} & =\beta_{0}+\left(\beta_{1}+\beta_{1Q}\sqrt{RQ_{t-1}^{\left(d\right)}}\right)RV_{t-1}^{\left(d\right)}+\beta_{2}RV_{t-1}^{\left(w\right)}+\beta_{3}RV_{t-1}^{\left(m\right)}+\beta_{4}VIX_{t-1}+e_{t}, \end{array}$$

where $VIX_{t-1}$ is the VIX value at time t−1. The β coefficients are the parameters to be estimated, and $e_{t}$ is the zero mean error term.

With the HAR, HARQ, and HARQ-X specifications introduced, attention must next be directed to the estimation procedures used to evaluate these models empirically. Given the diverse statistical characteristics of high-frequency realized variance, the choice of estimator determines the robustness and accuracy of the forecasting results. In this study, no additional constrains were applied for the estimation. Therefore, the smoothing phenomenon was out of scope when assessing relative volatility.

### 2.5. HAR Model Estimation and Evaluation

The OLS estimator commonly estimates the HAR model, where realized variance is the dependent variable. While it is a standard empirical application because of some well-known statistical properties of RV, OLS is not always the best choice. The stylized facts of realized variance, such as spikes/outliers, conditional heteroskedasticity, and non-Gaussianity, violate some of the most fundamental assumptions of the OLS model [25]. Therefore, OLS can only be considered the best estimator under the Gauss–Markov assumptions [63]. Consequently, OLS estimation results are inefficient, finitely sample-biased, and overly sensitive to outliers. Alternative estimation techniques such as Weighted Least Squares (WLS), Robust Linear Model (RLM), and Least Absolute Deviation (LAD) were studied in this work to address these limitations.

#### 2.5.1. Ordinary Least Squares

OLS is a linear regression method that estimates the parameters of a linear model by minimizing the sum of the squared differences between the observed and predicted values. For HAR model estimations, OLS finds β = ${\left(\beta_{1},\beta_{2}\ldots \beta_{n}\right)}^{⊺}$ coefficients by minimizing the Residual Sum of Squares (RSS) defined by Equation (20):

(20) $$\hat{\beta }=\arg\min_{\beta }\sum_{t=23}^{T}{\left(RV_{t}-\left(\beta_{0}+\beta_{1}RV_{t-1}^{\left(d\right)}+\beta_{2}RV_{t-1}^{\left(w\right)}+\beta_{3}RV_{t-1}^{\left(m\right)}\right)\right)}^{2},$$

where $RV_{t}$ is the realized variance at time *t*, and $RV_{t-1}^{\left(d\right)}$, $RV_{t-1}^{\left(w\right)}$, and $RV_{t-1}^{\left(m\right)}$ are the daily, weekly, and monthly realized variances at time t−1, respectively. The β coefficients are the parameters to be estimated. When considering asymptotic efficiency, OLS is the leading estimator for β, assuming the errors in autoregressions are independent, normally (Gaussian) distributed, and homoskedastic. Unfortunately, in financial volatility modeling, residuals are often neither normally distributed nor independent, and volatility shows heteroskedasticity. Therefore, while OLS can provide unbiased estimates under milder conditions, its efficiency and robustness are questionable in practice [63].

In this work, the logarithmic realized variance (logRV) served as both a proxy and a target for forecasting. There are several advantages to using logRV, one of which is avoiding negative values. Another positive feature is that it reduces skewness, making the data more symmetrical and closer to a normal distribution. Therefore, Equation (21) represents the reformulated minimization problem:

(21) $$\begin{array}{c} \hat{\beta }=\arg\min_{\beta }\sum_{t=23}^{T}{\left(\begin{array}{cc} \; & \log\left(RV_{t}\right)-(\beta_{0}+\beta_{1}\cdot \log\left(RV_{t-1}^{\left(d\right)}\right)+\\ \; & \beta_{2}\cdot \log\left(RV_{t-1}^{\left(w\right)}\right)+\beta_{3}\cdot \log\left(RV_{t-1}^{\left(m\right)}\right)+\\ \; & \beta_{4}\cdot \left(\log\left(RV_{t-1}\right)\cdot \log\left(RQ_{t-1}\right)\right)+\beta_{5}\cdot \log\left(VIX_{t-1}\right)) \end{array}\right)}^{2}, \end{array}$$

This minimization problem represents the estimation of an HARQ model with the exogenous variable VIX, where the dependent variable is the logarithm of the realized variance of day *t*, denoted as $\log\left(RV_{t}\right)$. The regressors include various lagged components, such as daily, weekly, and monthly realized variance terms—log$\left(RV_{t-1}^{\left(d\right)}\right)$, $\log\left(RV_{t-1}^{\left(w\right)}\right)$, and $\log\left(RV_{t-1}^{\left(m\right)}\right)$, respectively—that capture the heterogeneous memory of financial variance. The inclusion of the interaction term (log$\left(RV_{t-1}\right)\cdot \log\left(RQ_{t-1}\right)$) allows the model to adjust for measurement errors in the realized variance, as advocated by Bollerslev, Patton, and Quaedvlieg [22].

#### 2.5.2. Weighted Least Squares

Weighted Least Squares (WLS) is a regression technique that extends OLS by allowing for heteroskedasticity in the error terms. In WLS, each observation is assigned a weight, which can be used to correct for non-constant variance in the residuals. While OLS minimizes the sum of squared residuals (see Equation (22)), WLS minimizes the weighted sum of squared residuals, where each residual is scaled by a weight $w_{t}$ (see Equation (23)):

(22) $$\begin{array}{cc} \; & \hat{\beta }=\arg\min_{\beta }\sum_{t=23}^{T}{\left(RV_{t}-{\hat{RV}}_{t}\right)}^{2}, \end{array}$$

(23) $$\begin{array}{cc} \; & \hat{\beta }=\arg\min_{\beta }\sum_{t=23}^{T}w_{t}{\left(RV_{t}-\hat{RV_{t}}\right)}^{2}; \end{array}$$

where β represents the regression coefficients that this model minimizes, $w_{t}$ is the weight assigned to the observation at time *t*, and $\hat{RV_{t}}$ is the predicted value of the realized variance at time *t*. Such an approach helps reduce the influence of more volatile or noisier periods. For this reason, it is well suited for the HAR model and can be defined by Equation (24):

(24) $$\begin{array}{c} \hat{\beta }=\arg\min_{\beta }\sum_{t=23}^{T}w_{t}{\left(\begin{array}{cc} \; & \log\left(RV_{t}\right)-(\beta_{0}+\beta_{1}\cdot \log\left(RV_{t-1}^{\left(d\right)}\right)+\\ \; & \beta_{2}\cdot \log\left(RV_{t-1}^{\left(w\right)}\right)+\beta_{3}\cdot \log\left(RV_{t-1}^{\left(m\right)}\right)+\\ \; & \beta_{4}\cdot \left(\log\left(RV_{t-1}\right)\cdot \log\left(RQ_{t-1}\right)\right)+\beta_{5}\cdot \log\left(VIX_{t-1}\right)) \end{array}\right)}^{2}, \end{array}$$

where $w_{t}$ is the weight assigned to the observation at time *t*. The WLS estimator outperforms the OLS estimator if each weight ($w_{t}$) is inversely proportional to the conditional variance of the associated error ($e_{t}$). In this approach, errors that are likely to be significant weigh less. Although Patton and Sheppard employed a straightforward WLS approach to estimate the HAR model, they did not consider other options [19].

#### 2.5.3. Least Absolute Deviations

The Least Absolute Deviation (LAD) estimator is another robust alternative to the Ordinary Least Squares (OLS) method for linear regression analysis. Whereas OLS minimizes the sum of the squared residuals, LAD minimizes the sum of the absolute residuals and is therefore less sensitive to outliers in the dependent variable [64].

Describing explanatory variables as vector $X_{t-1}$ (see Equation (25)), estimation coefficients as β (see Equation (27)), and the dependent variable as $y_{t}$ (see Equation (26)), we have:

(25) $$\begin{array}{ccc} X_{t-1} & =\left(\begin{array}{c} 1,\log\left(RV_{t-1}^{\left(d\right)}\right),\log\left(RV_{t-1}^{\left(w\right)}\right),\log\left(RV_{t-1}^{\left(m\right)}\right),\\ \left(\log\left(RV_{t-1}\right)\cdot \log\left(RQ_{t-1}\right)\right),\log\left(VIX_{t-1}\right) \end{array}\right), & \;X_{t-1}\in R^{k} \end{array}$$

(26) $$\begin{array}{ccccccccccccc} y_{t} & =\log\left(RV_{t}\right),\; & & & & & & & & & & & \;y_{t}\in R^{k} \end{array}$$

(27) $$\begin{array}{cccccccccccc} \beta & ={\left(\beta_{1},\beta_{2}\ldots \beta_{n}\right)}^{⊺};\; & & & & & & & & & & \;\beta \in R^{k} \end{array}$$

where *k* is the number of explanatory variables, $y_{t}$ is the dependent variable, and $X_{t-1}$ is the vector of explanatory variables at time t−1. The LAD estimator $\hat{\beta }$ is defined as the solution to the optimization problem described in Equation (28):

(28) $$\hat{\beta }=\arg\min_{\beta }\sum_{t=23}^{T}\left|y_{t}-X_{t-1}\cdot \beta \right|,$$

where $X_{t-1}$ is the vector of explanatory variables at time t−1, and $y_{t}$ is the dependent variable at time *t*. This equation can be transformed and used as Equation (29):

(29) $$\hat{\beta }=\arg\min_{\beta }\sum_{t=23}^{T}\tau \left(y_{t}-X_{t-1}\cdot \beta \right),$$

where 0<τ<1 in quantile regression represents the intended quantile level estimation. Let $u_{t}$ be the error; then, it can be described as Equation (30):

(30) $$u_{t}=y_{t}-X_{t-1}\cdot \beta ,$$

where $u_{t}$ is the error term at time *t*. When τ=0.5, it is referred to as median regression, which is equivalent to LAD regression. Therefore, in this implementation, τ=0.5 was used for quantile regression. The mathematical representation of the absolute value function τ(u) is defined as Equation (31). This function is used to calculate the absolute error in the LAD regression.

(31) $$\begin{array}{cc} \tau (u) & =\left|u\right|=\left\{\begin{array}{cc} u, & \mathrm{if}\;u\geq 0\\ -u, & \mathrm{otherwise}\; \end{array}\right. \end{array},$$

where *u* is the error term at time *t*. The linear and symmetric penalty of the absolute function accounts for both overestimates and underestimates. As there are outliers and a heavy-tailed error distribution, which is common for financial time series, this feature of LAD enhances its robustness. However, due to its non-differentiability at zero, LAD estimation typically requires optimization techniques such as linear programming. Despite this, its resilience to extreme values makes it especially suitable for volatility modeling and robust econometric forecasting.

#### 2.5.4. Robust Regression Models

Although OLS is a good option in ideal circumstances, in real life, unideal conditions make this model extremely sensitive to outliers or odd observations in the data. More reliable estimators, such as the widely used M-estimator introduced by Huber, have thus been suggested as substitutes [65]. The M-estimator of β solves the HAR model’s minimization problem and is defined by Equation (32):

(32) $$\hat{\beta }=\arg\min_{\beta }\sum_{t=23}^{T}\rho \left(y_{t}-X_{t-1}\cdot \beta \right),$$

where ρ is a symmetric function that has a unique minimum at zero and is predefined, $y_{t}$ is the dependent variable at time *t*, and $X_{t-1}$ is the vector of explanatory variables at time t−1. In the presence of outliers or heteroskedastic errors, RLM is a powerful tool to estimate coefficients. It represents the idea of minimizing a robust objective function. The Minkowski loss function [66,67], defined in Equation (33), provides a flexible and powerful alternative to the traditional loss functions used in the regression analysis employed in this research. Thus, it was used to estimate the model when applied as a custom function in this study.

(33) $$L=\sum_{t=23}^{T}{\left|RV_{t}-\hat{RV_{t}}\right|}^{p},$$

where *L* is the loss function, $RV_{t}$ is the realized variance at time *t*, $\hat{RV_{t}}$ is the predicted value of the realized variance at time *t*, and *p* is the sensitivity parameter of the Minkowski distance. One of the advantages of the Minkowski loss function is that it generalizes both the Least Absolute Deviation (LAD) and Ordinary Least Squares (OLS) approaches by allowing the exponent *p* to be tuned. As seen from Equation (33), the choice of *p* directly influences the estimator’s sensitivity to outliers and the tail behavior of the error distribution. There is a trade-off between robustness and efficiency in this process. When p=1, this function is as sensitive as the LAD (Manhattan norm); when p=2, this function acts like the OLS (Euclidean norm) estimator. Otherwise, when 1<p<2, this function becomes more sensitive to outliers than LAD but less sensitive than OLS, which means the Minkowski loss reduces the influence of extreme observations without completely disregarding them. Fractional values of *p*, such as p=1.2 or p=1.4, offer a balance that avoids instability or inefficiency problems. These features make it especially valuable for financial time series analysis.

The continuous nature of the Minkowski norm also enables fine-tuned control over the estimator’s properties, something that is hard to achieve using discrete robust loss functions such as Huber’s or Tukey’s bi-weight. This tunability enables practitioners to calibrate the model more precisely to the data’s structure, thereby enhancing both forecast accuracy and model reliability. For the implementation of this method in this work, the minimization problem can be described by Equation (34):

(34) $$\hat{\beta }=\arg\min_{\beta }\sum_{t=23}^{T}{\left|y_{t}-X_{t-1}\cdot \beta \right|}^{p},$$

where $y_{t}$ is the dependent variable at time *t*, $X_{t-1}$ is the vector of explanatory variables at time t−1, and β is the vector of coefficients to be estimated.

#### 2.5.5. Entropy Loss Function

The Entropy Loss Function (ELF) is a robust alternative to the traditional loss functions used in regression analysis. To estimate the HAR model coefficients, an alternative approach—the Entropy Loss Function [68,69]—was also applied. The Entropy Loss Function (ELF) applies both squared and relative error penalties. The ELF is defined by Equation (35), which minimizes the entropy of the residuals:

(35) $$\hat{\beta }=\arg\min_{\beta }L=\arg\min_{\beta }\sum_{t=23}^{T}\left[\frac{{\left(y_{t}-{\hat{y}}_{t}\right)}^{2}\cdot \left(1-K_{exp}\right)}{2\cdot y_{t}^{2}}+\frac{{\left(y_{t}-{\hat{y}}_{t}\right)}^{2}\cdot K_{exp}}{2}\right],$$

where *L* is the loss function, $y_{t}$ is the actual value of the logarithmic RV at time *t*, $\hat{y_{t}}$ is the predicted value of the dependent variable at time *t*, and $K_{exp}$ is a user-defined constant weighting parameter governing the trade-off between relative and absolute error penalization. The assumption of this study is that $y_{t}=RV_{t}$ and $\hat{y_{t}}=\hat{RV_{t}}$. Rearranging this equation results in Equation (36):

(36) $$L_{t}=\frac{{\left(y_{t}-\hat{y_{t}}\right)}^{2}}{2}\cdot \left(\frac{1-K_{exp}}{y_{t}^{2}}+K_{exp}\right),$$

where $L_{t}$ is the loss function, $y_{t}$ is the actual value of the logarithmic RV at time *t*, $\hat{y_{t}}$ is the predicted value of the dependent variable at time *t*, and $K_{exp}$ is a user-defined constant weighting parameter governing the trade-off between relative and absolute error penalization.

**Figure 5:**
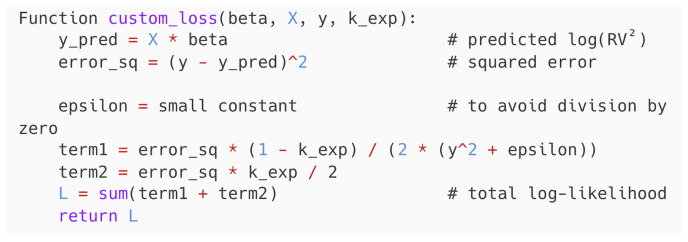
Pseudo-code of the custom Entropy Loss Function.

**Figure 6:**
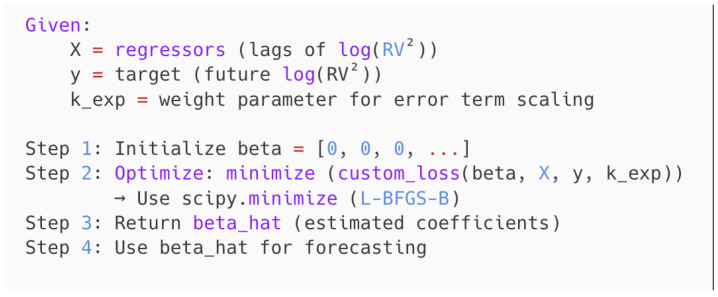
Pseudo-code of the estimation procedure using custom loss.

After rearranging the equation with the variables defined for this work, the minimization problem can be described by Equation (37):

(37) $$\hat{\beta }=\arg\min_{\beta }L=\arg\min_{\beta }\sum_{t=23}^{T}\left[\frac{{\left(y_{t}-X_{t-1}\cdot \beta \right)}^{2}}{2}\cdot \left(\frac{1-K_{exp}}{y_{t}^{2}}+K_{exp}\right)\right],$$

where $y_{t}$ is the dependent variable at time *t*, $X_{t-1}$ is the vector of explanatory variables at time t−1, β is the vector of coefficients to be estimated, and $K_{exp}$ is a user-defined constant weighting parameter governing the trade-off between relative and absolute error penalization. Figure 5 and Figure 6 illustrate the pseudo-code of the definition and the implementation of the Entropy Loss Function, respectively.

The parameter $K_{exp}\in \left[0,1\right]$ determines the trade-off between the relative loss when $K_{exp}\to 0$ and the squared error loss when $K_{exp}\to 1$. When applied to volatility modeling, the ELF allows the model to remain sensitive to variance magnitudes while avoiding excessive penalization of large-scale fluctuations. The proposed method penalizes both the absolute and proportional deviations in a unified manner. As a result, the coefficient estimation process enhances both robustness and accuracy.

#### 2.5.6. Summary

To summarize, the estimation techniques used in Section 2.5 have been employed to fit HAR-type models, emphasizing the need for robustness in the presence of financial data irregularities, such as outliers and heteroskedasticity. While OLS is a standard technique for this problem, it performs inaccurately due to its sensitivity to non-Gaussian errors. To address this limitation, two different approaches—WLS and LAD—were applied. Furthermore, the introduced Minkowski loss function balanced out the OLS and LAD. Additionally, an entropy-based loss function was applied, which also balanced absolute and relative error penalties. These techniques collectively strengthen the predictive power and resilience of HAR models under real-world market conditions.

### 2.6. Forecast Design

In this section, the forecasting procedure is discussed, including rolling window forecasting and multi-horizon forecast targets. The forecasting aims to generate out-of-sample predictions of future log realized variance.

#### 2.6.1. Rolling Window Forecasting

The applied rolling window forecasting evaluates a model’s genuine out-of-sample forecasting. In this approach, the model uses the latest available data each time and also prevents possible look-ahead bias. The forecasting procedure operates by estimating the model using a fixed-size training window consisting of the most recent 1000 daily observations at each iteration. Then, the loss function obtains model parameters. Such estimated coefficients generate a single or multi-step forecast of realized variance for the corresponding target horizon. Finally, following each forecast, the window is rolled forward by one day by discarding the oldest observation and including the newest one. This process continues until the entire out-of-sample evaluation period passes for forecasting. The result is a sequence of rolling out-of-sample predictions. Each forecast uses only past data, which makes them directly comparable to observed actual realized variance values. This framework reflects how the model would work in real-time scenarios.

#### 2.6.2. Forecasting Targets

The designed forecasting framework predicts the future value of the logarithm of realized variance. To reflect different investment and risk management perspectives, the resulting forecasts take multiple time horizons into account. This study set four distinct forecast targets for evaluation, namely, daily (D=1), weekly (D=5), biweekly (D=10), and monthly (D=22). The logarithmic scale of cumulative realized variance expresses each of the targets described in Equation (38):

(38) $$y_{t}^{D}=\log\sum_{j=1}^{D}RV_{t+j},$$

where $y_{t}^{D}$ is the target variable at time *t* for horizon *D*, and $RV_{t+j}$ is the forecasted realized variance at time t+j. For the empirical practice of HAR-type models, the abovementioned targets are common. To ensure compatibility with the regression framework and preserve the positive nature of the forecast variable, both actual realized variance and forecasted variance were log-transformed. The log transformation of the model features stabilized variance and improved the distributional properties of the model residuals. The non-overlapping rolling sums composed the target variables in order to maintain clarity and reduce autocorrelation in the forecast errors across horizons.

### 2.7. Evaluation Metrics

This study employed two performance metrics for the assessment of the forecasting results: Mean Absolute Error (MAE) and Quasi-Likelihood (QLIKE) loss. These metrics ensured that the evaluation was fair, unbiased, and methodologically rigorous. The Mean Absolute Error (MAE) (see Equation (10)) measures the average absolute deviation between the forecasted and actual values of log-realized variance. One of the main advantages of the MAE is that it is independent of model training under some specific objective function. This advantage means that models fitted with different estimation strategies are comparable on an equal basis. Another reason for using the MAE is that it is robust to outliers due to its linear penalization of error magnitudes, which is also important given the nature of financial time series. Since the target variable is on a log scale, the MAE provides a direct error measurement within the same domain, maintaining coherence with the regression specification.

(39) $$MAE=\frac{1}{n}\sum_{t=1}^{n}\left|\log RV_{t}-\hat{\log RV_{t}}\right|,$$

where *n* is the number of observations, $\log RV_{t}$ is the actual value of log-realized variance at time *t*, and $\hat{\log RV_{t}}$ is the forecasted value of log-realized variance at time *t*.

The Quasi-Likelihood (QLIKE) loss provides a likelihood-based evaluation criterion tailored to variance forecasting. The derivation of the Gaussian Quasi-Likelihood function is the QLIKE function, which is known to be asymptotically optimal under conditional heteroskedasticity. Its key advantage is its asymmetric penalty structure. Suppose the variance under-prediction is penalized more severely than over-prediction. That makes it well aligned with risk-sensitive financial applications. For example, QLIKE is particularly relevant when model outputs aid in estimating risk management, portfolio allocation, or Value-at-Risk (VaR) estimation. Although the models use and predict variance in log space, QLIKE cannot be directly applied to logRV forecasts and actuals. Therefore, the regression and forecast are in logRV; thus, to compute QLIKE, they must be changed back (Equations (40) and (41)) as follows:

(40) $$\begin{array}{cc} RV_{actual} & =\exp\left(\log RV_{t}\right), \end{array}$$

(41) $$\begin{array}{cc} RV_{forecast} & =\exp\left(\hat{\log RV_{t}}\right); \end{array}$$

where $RV_{actual}$ is the actual value of realized variance at time *t*, and $RV_{forecast}$ is the forecasted value of realized variance at time *t*. After this transformation, the formula for QLIKE can be expressed as Equation (42):

(42) $$QLIKE=\frac{RV_{actual}^{2}}{RV_{forecast}^{2}}-\log\left(\frac{RV_{actual}^{2}}{RV_{forecast}^{2}}\right)-1,$$

where $RV_{actual}$ is the actual value of realized variance at time *t*, and $RV_{forecast}$ is the forecasted value of realized variance at time *t*.

Together, MAE and QLIKE maintain a balance between robustness, interpretability, and theoretical efficiency. MAE ensures fairness across models, regardless of the estimation strategy [70], while QLIKE provides a probabilistically grounded criterion sensitive to variance estimation accuracy. Their joint application supports a comprehensive and equitable evaluation framework for comparing the predictive performance of HAR-type models across different forecast horizons and estimation methods.

## 3. Results and Discussion

This section presents the empirical results of the HAR-type models examined in this study. Three different extensions of the HAR model were considered, namely, HAR-RV, HARQ, and HARQ-X, each with five different estimators used for performance comparison. The out-of-sample forecasting performance evaluation, using four horizons—daily, weekly, biweekly, and monthly—was based on aggregated realized variance. The parameter *p* of the RV estimator was tuned via exhaustive search to demonstrate the models’ ability to achieve error minimization for different error criteria with different *p* values.

Table 3 presents a comparative analysis of the HAR-RV model with the estimators OLS, LAD, WLS, RLM, and ELF. According to the results, the primary contenders (the cell values highlighted in **bold**) for this model were the RLM and entropy estimation techniques. While the Entropy Loss Function consistently achieved the lowest QLIKE values across all forecasting horizons, suggesting its superiority in capturing variance dynamics, RLM with a custom Minkowski loss function (p=1.3) demonstrated its accuracy in the MAE metric. Although the entropy estimator did not exhibit the best MAE performance, its results were not significantly different from those of other estimators. In contrast, this was not true for the RLM (p=1.3) technique. Given the consistently strong performance of the entropy-based model in minimizing the QLIKE loss and achieving significant results in the MAE metric, the entropy technique is a robust alternative to conventional estimation techniques within the HAR-RV framework. On the other hand, the WLS technique delivered the best MSE values, which is not surprising, considering that it explicitly minimizes the weighted Mean Squared Error in the optimization problem.

Table 4 reflects relatively similar results, comparing the exact applied estimators with the same conditions applied for the HARQ model. Likewise, in this instance, the ELF demonstrated the best performance in the QLIKE metric due to its alignment with logarithmic likelihood-based forecasting. Furthermore, the RLM with a custom Minkowski loss function (p=1.3) estimator delivered optimal MAE results for all forecasting horizons. The WLS approach with RQ weight performed better, as indicated by MSE values similar to the previous investigation. In this experiment, the Entropy Loss Function resulted in the same magnitude of MAE as other estimation techniques. Therefore, these results lead to the conclusion that the Entropy Loss Function is well suited for the HARQ model.

The evaluation of the HARQ-X model was conducted under the same conditions, with one exception—the introduction of an additional coefficient in the Entropy Loss Function ($K_{exp}=0.01$). The results in Table 5 exhibit the superiority of ELF in minimizing the QLIKE metric. In contrast, the RLM (p=1.3) showed the lowest MAE for daily and monthly horizons, while LAD exhibited the lowest MAE for weekly and biweekly horizons. This experiment presented the lowest Entropy Loss Function results in MAE compared with other models, especially in longer forecasting horizons. Conversely, the Robust Linear Model with a custom Minkowski loss function (p=2.1) yielded balanced trade-off results on both metrics. The WLS approach with RQ weight demonstrated the lowest MSE values with this model as well. Conversely, the Robust Linear Model with a custom Minkowski loss function (*p* = 2.1) yielded balanced trade-off results on all the metrics.

Overall, the results in Table 3, Table 4 and Table 5 suggest that introducing more lag to HAR-type models improves their accuracy. Due to the differences in each model optimization problem for these techniques, some of them demonstrated superior forecasting performance against others. For example, the WLS technique generally outperformed other models across all horizons, as measured using the MSE criterion. Although LAD primarily minimized the MAE metric in its optimization problem, it was outperformed by RLM with a custom Minkowski loss after tuning the *p*-value, demonstrating the flexibility of the latter approach. The Entropy Loss Function showed the best performance in the QLIKE metric compared to other models and techniques used in this work, reflecting its strong alignment with the underlying distributional characteristics of variance forecasts.

**Figure 7:**
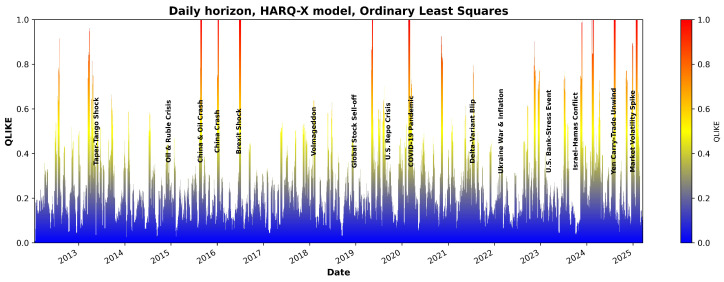
QLIKE estimates using the HARQX model with ordinary least squares for the daily horizon.

**Figure 8:**
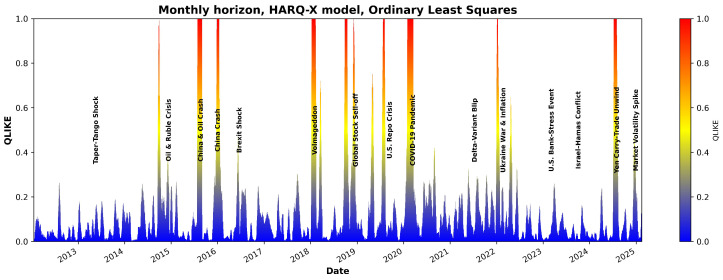
QLIKE estimates using the HARQX model with ordinary least squares for the monthly horizon.

Figure 7 and Figure 8 illustrate the QLIKE error metric for the HARQ-X model estimated with OLS over the entire period analyzed. As the visualization in Figure 7 and Figure 8 demonstrate, the model’s forecast accuracy significantly deteriorates during periods of high market stress. These periods of poor performance, indicated by spikes in the QLIKE metric, align closely with major market events that have been labeled on the chart for comparison. One of the candidate examples is the sharp increase in forecast error during the COVID-19 shock in early 2020, but other marked events also show a clear impact on the model’s predictive power. Although a detailed causal analysis of each individual error spike is beyond the scope of this study, this visualizes the model’s primary failure points, demonstrating its sensitivity to the types of structural breaks represented by the labeled events.

**Figure 9:**
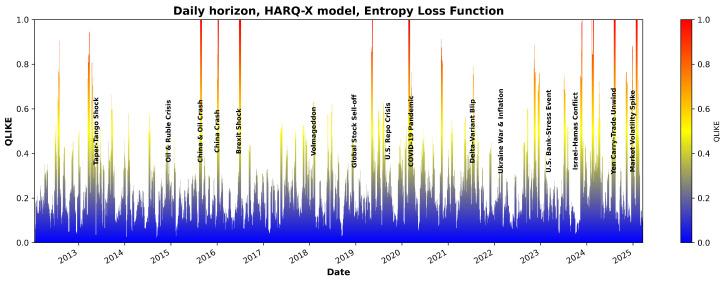
QLIKE estimates using the HARQX model with entropy loss for the daily horizon.

**Figure 10:**
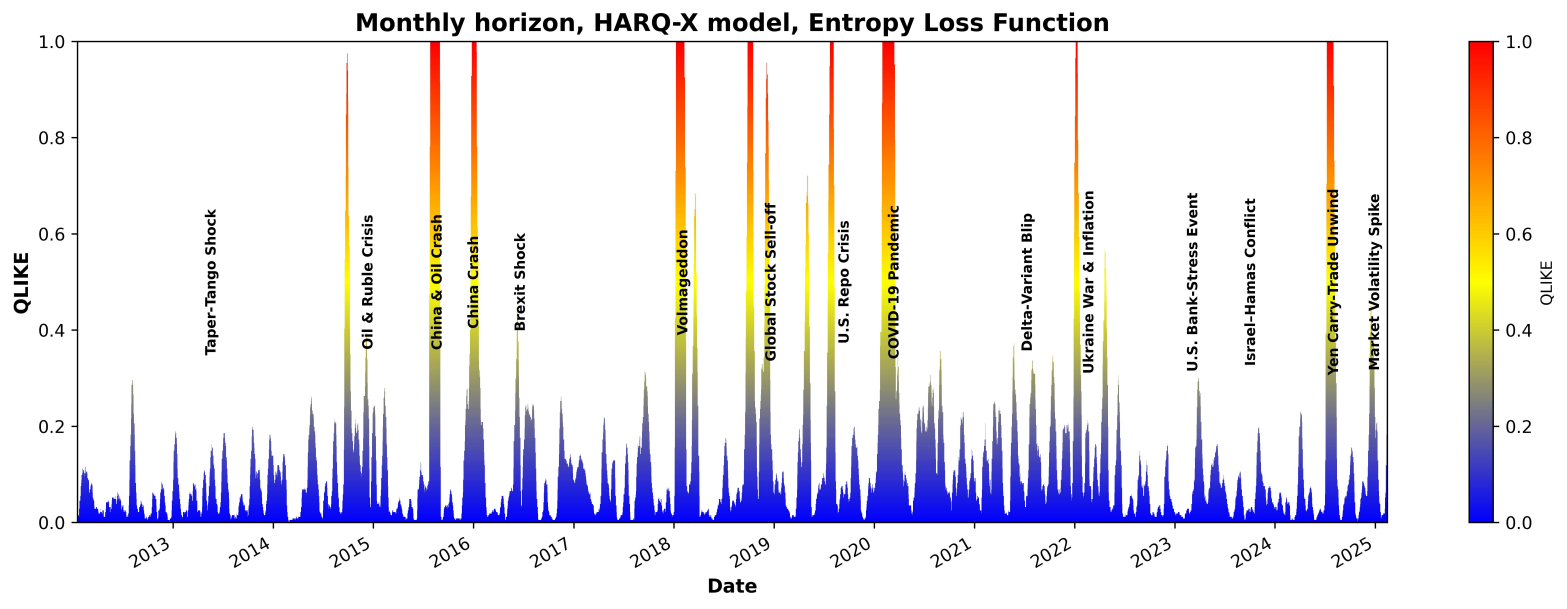
QLIKE estimates using the HARQX model with entropy loss for the monthly horizon.

Despite producing better overall average results, the effect of the entropic loss function (as shown in Figure 9 and Figure 10) suggests that the estimation model infrastructure and constraints require further investigation.

**Figure 11:**
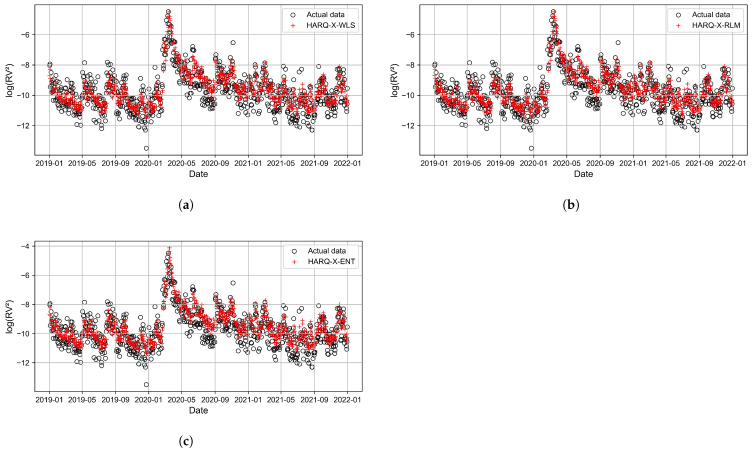
Best-performing HARQ-X models for the daily horizon, where (**a**) represents the WLS approach, (**b**) represents the RLM (p=1.3) approach, and (**c**) represents the ELF approach.

**Figure 12:**
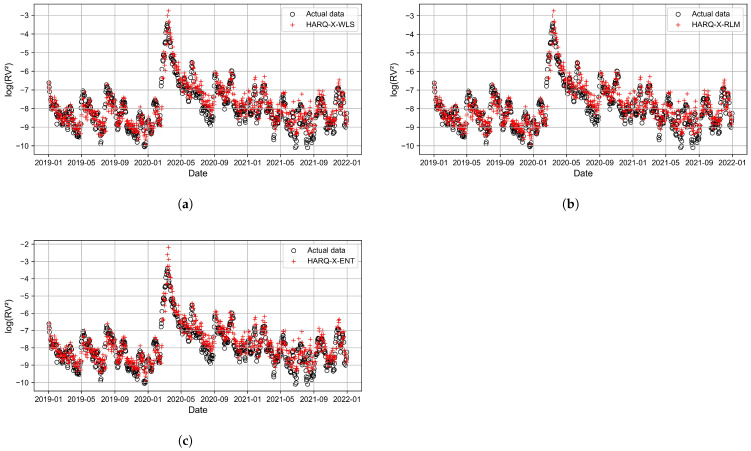
Best-performing HARQ-X models for the weekly horizon, where (**a**) represents the WLS approach, (**b**) represents the RLM (p=1.3) approach, and (**c**) represents the ELF approach.

**Figure 13:**
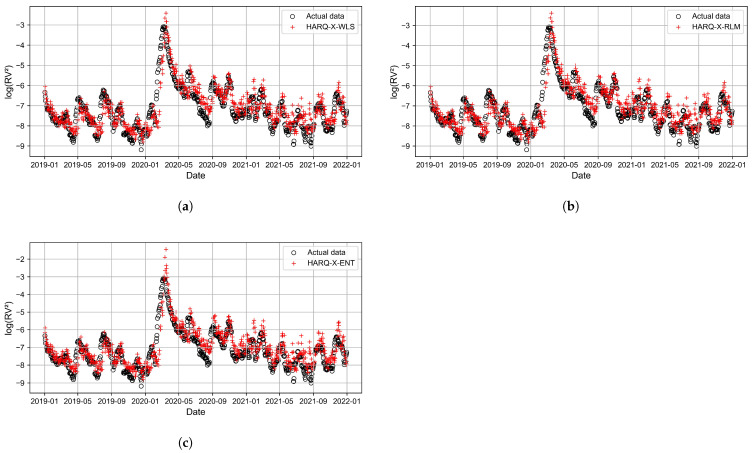
Best-performing HARQ-X models for the biweekly horizon, where (**a**) represents the WLS approach, (**b**) represents the RLM (p=1.3) approach, and (**c**) represents the ELF approach.

**Figure 14:**
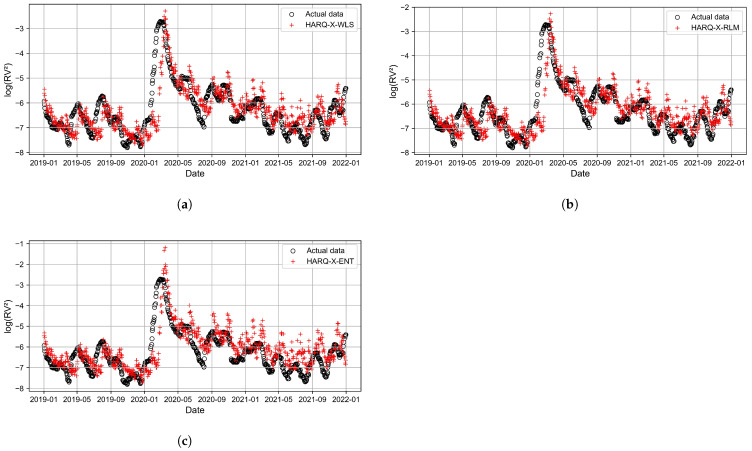
Best-performing HARQ-X models for the monthly horizon, where (**a**) represents the WLS approach, (**b**) represents the ELF approach, and (**c**) represents the RLM (p=1.3) approach.

Figure 11, Figure 12, Figure 13 and Figure 14 present the time series plots of the HARQ-X model with daily, weekly, biweekly, and monthly horizons, respectively. These figures show only the optimal results generated by three estimation techniques: WLS (Figure 11a, Figure 12a, Figure 13a, Figure 14a), ELF (Figure 11b, Figure 12b, Figure 13b, Figure 14b), and RLM (*p* = 1.3, Figure 11c, Figure 12c, Figure 13c, Figure 14c). The specially selected timeline, from January 2019 to January 2022, better represents the models’ performance and actual values of the logarithmic realized variance. Additionally, during that period, stock markets crashed due to COVID-19. This period of increased volatility allowed us to observe volatility spikes and forecasting results.

## 4. Conclusions

In conclusion, this study comprehensively explored HAR-type models, including the Heterogeneous Autoregressive for Realized Variance (HAR-RV), the Heterogeneous Autoregressive Model with Realized Quarticity (HARQ), and the Heterogeneous Autoregressive Model with Realized Quarticity extension incorporating an exogenous variable, VIX (HARQ-X). Generally, the observed forecasting models showed that the performance of the HAR-type models improved when extended with the realized quarticity and an exogenous variable (VIX). Moreover, this research presented five distinctive HAR-type model coefficient estimation techniques to suggest an alternative approach to traditional methods. The presented techniques were Ordinary Least Squares (OLS), Least Absolute Deviation (LAD), Weighted Least Squares (WLS), Robust Linear Model (RLM) with a custom Minkowski function, and Entropy Loss Function (ELF).

The forecasting accuracy evaluation took place over several forecasting periods, including daily (1 D), weekly (5 D), biweekly (10 D), and monthly (22 D). With the availability of high-frequency SPX stock market index data, the calculated realized variance acted as both a model feature and a target. Additionally, the coefficients estimated by the different techniques, including the Mean Absolute Error (MAE), the Mean Squared Error (MSE), and the Quasi-Likelihood (QLIKE) coefficients, were evaluated and compared to assess model performance. The empirical results of this study revealed that the ELF performed best when evaluating the coefficients of the HAR-RV and HARQ models, showing the lowest QLIKE coefficient and decent MAE and MSE results. Moreover, the RLM technique exhibited the least MAE while showing decent results for QLIKE and MSE. The HARQ-X model demonstrated the best performance in terms of QLIKE coefficients when estimated with the ELF. The RLM with a custom Minkowski loss function (p=1.3) yielded the lowest MAE for daily and monthly horizons, while LAD exhibited the lowest MAE for weekly and biweekly horizons. The Entropy Loss Function results in this experiment showed the lowest MAE compared to other models, especially in longer forecasting horizons. The WLS approach with the realized quarticity weight factor obtained the best MSE results; conversely, the Robust Linear Model with a custom Minkowski loss function (p=2.1) yielded balanced trade-off results on all the metrics.

## Figures and Tables

**Table 1:** SPX 5 min raw data snapshot.

Datetime	Open	High	Low	Close
2008-01-02 09:35:00	1470.17	1470.17	1467.88	1469.49
2008-01-02 09:40:00	1469.78	1471.71	1469.39	1471.22
2008-01-02 09:45:00	1471.56	1471.77	1470.69	1470.78
2008-01-02 09:50:00	1470.28	1471.06	1470.1	1470.74
2008-01-02 09:55:00	1470.81	1470.81	1468.42	1469.37

**Table 2:** Intraday frequencies and subinterval counts (*M*).

Interval Length (Δ)	Subintervals per Day	Notes
1 min	390	Ultra-high frequency
2 min	195	
3 min	130	
5 min	78	Standard for RV computation
10 min	39	
15 min	26	Lower frequency, less detail
30 min	13	
60 min (1 h)	6.5	Usually rounded to 6 or 7
195 min	2	Half-day intervals
390 min	1	One daily observation (EOD)

**Table 3:** HAR-RV model’s (Equation (16)) out-of-sample forecasting results using different estimation techniques.

	OLS	LAD	WLS_RQ_	RLM *p* = 2.1	RLM *p* = 1.3	ELF $K_{exp}$ = 0.1
**Daily horizon**						
QLIKE	0.342028	0.345926	0.342150	0.341260	0.345827	**0.321536**
MAE	0.579774	0.579553	0.579665	0.579930	**0.579114**	0.580111
MSE	0.541348	0.541339	0.541206	0.541537	**0.540675**	0.541350
**Weekly horizon**						
QLIKE	0.166367	0.177811	0.166370	0.165485	0.173596	**0.165114**
MAE	0.384559	0.382425	0.384370	0.385148	**0.382333**	0.385893
MSE	0.251494	0.255576	**0.251327**	0.251533	0.253271	0.251493
**Biweekly horizon**						
QLIKE	0.227243	0.248310	0.227262	0.227243	0.241540	**0.223707**
MAE	0.423649	0.421023	0.423434	0.424573	**0.420693**	0.426678
MSE	0.318779	0.326519	**0.318584**	0.318715	0.322992	0.318777
**Monthly horizon**						
QLIKE	0.370091	0.436770	0.370073	0.365128	0.413238	**0.359545**
MAE	0.464938	0.459841	0.464795	0.467163	**0.458579**	0.473115
MSE	0.397698	0.419242	**0.397519**	0.397602	0.409113	0.397699

**Table 4:** HARQ model’s (Equation (18)) out-of-sample forecasting results using different estimation techniques.

	OLS	LAD	WLS_RQ_	RLM *p* = 2.1	RLM *p* = 1.3	ELF $K_{exp}$ = 0.1
**Daily horizon**						
QLIKE	0.308979	0.323848	0.308999	0.307671	0.319208	**0.306458**
MAE	0.543546	0.542170	0.5434	0.543920	**0.541912**	0.544287
MSE	0.478849	0.481396	**0.478685**	0.479025	0.479668	0.479030
**Weekly horizon**						
QLIKE	0.155273	0.165662	0.155253	0.154416	0.162043	**0.154245**
MAE	0.366248	0.364350	0.366114	0.366870	**0.364036**	0.367386
MSE	0.231442	0.235199	**0.231280**	0.231505	0.233154	0.231651
**Biweekly horizon**						
QLIKE	0.218227	0.239794	0.218224	0.216364	0.233033	**0.215052**
MAE	0.543546	0.542170	0.5434	0.543920	**0.541912**	0.544287
MSE	0.301743	0.309658	**0.301538**	0.301734	0.305979	0.302221
**Monthly horizon**						
QLIKE	0.366199	0.431019	0.366162	0.361283	0.409767	**0.356297**
MAE	0.458733	0.453891	0.458593	0.460855	**0.452515**	0.466573
MSE	0.389303	0.409066	**0.389112**	0.389269	0.400158	0.390595

**Table 5:** HARQ-X model’ (Equation (19)) out-of-sample forecasting results using different estimation techniques.

	OLS	LAD	WLS_RQ_	RLM *p* = 2.1	RLM *p* = 1.3	ELF $K_{exp}$ = 0.1	ELF $K_{exp}$ = 0.01
**Daily horizon**							
QLIKE	0.278937	0.290429	0.278922	0.277805	0.287298	0.276995	**0.268285**
MAE	0.522870	0.523373	0.522898	0.523026	**0.522701**	0.523440	0.526647
MSE	0.446430	0.448930	0.446519	**0.446398**	0.447735	0.446812	0.449631
**Weekly horizon**							
QLIKE	0.132194	0.138245	0.132158	0.131710	0.136197	0.131713	**0.129863**
MAE	0.345263	**0.339749**	0.345213	0.346180	0.340703	0.346390	0.350214
MSE	0.204014	0.203218	0.203967	0.204422	**0.202857**	0.204676	0.206861
**Biweekly horizon**							
QLIKE	0.190002	0.206361	0.189978	0.188780	0.200254	0.188493	**0.184266**
MAE	0.384005	**0.376994**	0.383951	0.385496	0.377558	0.386880	0.396262
MSE	0.265001	0.266835	0.264918	0.265749	**0.264141**	0.267138	0.274253
**Monthly horizon**							
QLIKE	0.337472	0.381946	0.337457	0.333633	0.367668	0.331365	**0.319398**
MAE	0.427724	0.417575	0.427714	0.430771	**0.417262**	0.437475	0.469005
MSE	0.353712	0.362413	**0.353673**	0.354721	0.356853	0.360805	0.389078

## Data Availability

The data presented in this study are available on request from the corresponding author.

## Abbreviations

The following abbreviations are used in this manuscript:

ANN	Artificial Neural Network
ARCH	Autoregressive Conditional Heteroskedasticity
ARIMA	Autoregressive Integrated Moving Average
Bi-RNN	Bidirectional Recurrent Neural Network
CNN	Convolutional Neural Network
ECON	Economics
EGARCH	Exponential Generalized Autoregressive Conditional Heteroskedasticity
ELF	Entropy Loss Function
ESN	Echo State Neural Network
GARCH	Generalized Autoregressive Conditional Heteroskedasticity
GBM	Geometric Brownian Motion
GBP	(GRU–BiRNN–PSO)
GJR-GARCH	Glosten–Jagannathan–Runkle Generalized Autoregressive Conditional Heteroskedasticity
GRU	Gated Recurrent Unit
HAR	Heterogeneous Autoregressive
HARQ	Heterogeneous Autoregressive model with Realized Quarticity
HAR-RV	Heterogeneous Autoregressive model of Realized Volatility
HAR-RV-CJ	Heterogeneous Autoregressive model of Realized Variance with Continuous and Jump components
HARQ-X	Heterogeneous Autoregressive Model with realized Quarticity and Exogenous Variables
IQ	Integrated Quarticity
LAD	Least Absolute Deviation
LASSO	Least Absolute Shrinkage and Selection Operator
LSTM	Long Short-Term Memory
LST-HAR	Logistic Smooth Transition HAR
MAE	Mean Absolute Error
ML	Machine Learning
MT-GARCH	Multi-Task Generalized Autoregressive Conditional Heteroskedasticity
MTL-GARCH	Multi-Task Learning Generalized
NARX	Nonlinear Autoregressive with Exogenous Input
NBEATSx	Neural Basis Expansion Analysis for Time Series with Exogenous Variables
NLP	Natural Language Processing
OLS	Ordinary Least Squares
PCA	Principal Component Analysis
PSO	Particle Swarm Optimization
QLIKE	Quasi-Likelihood Loss
RF	Random Forest
RLM	Robust Linear Model
RQ	Realized Quarticity
RV	Realized Variance
SPX	Standard & Poor’s 500
SV	Stochastic Volatility
SVM	Support Vector Machine
TCN	Temporal Convolutional Network
V-HAR	Vector HAR
VIX	Chicago Board Options Exchange Volatility
VMD	Variational Mode Decomposition
WLS	Weighted Least Squares
